# Changes in plasma biomarkers following treatment with cabozantinib in metastatic castration-resistant prostate cancer: a post hoc analysis of an extension cohort of a phase II trial

**DOI:** 10.1186/s12967-015-0747-y

**Published:** 2016-01-13

**Authors:** Raya Leibowitz-Amit, Melania Pintilie, Leila Khoja, Arun A. Azad, Raanan Berger, A. Douglas Laird, Dana T. Aftab, Kim N. Chi, Anthony M. Joshua

**Affiliations:** Department of Oncology, Sheba Medical Center, Tel Hashomer, Israel; Division of Biostatistics, Princess Margaret Cancer Center, University Health Network, Toronto, Canada; Division of Medical Oncology and Hematology, Princess Margaret Cancer Center, University Health Network, 610 University Ave, Toronto, ON M5G 2M9 Canada; Department of Medical Oncology, British Columbia Cancer Agency, Vancouver, British Columbia, Canada; Exelixis, Inc, South San Francisco, CA USA

**Keywords:** Prostate cancer, Cabozantinib, Biomarker, c-MET, VEFR, VEGF

## Abstract

**Background:**

Cabozantinib is an orally available inhibitor of tyrosine kinases including VEGFR2 and c-MET. We performed a post hoc analysis to find associations between select plasma biomarkers and treatment response in patients (pts) with metastatic castration resistant prostate cancer (mCRPC) who received cabozantinib 100 mg daily as part of a phase 2 non-randomized expansion cohort (NCT00940225).

**Methods:**

Plasma samples were collected at baseline, 6 weeks and at time of maximal response from 81 mCRPC pts with bone metastases, of which 33 also had measurable soft-tissue disease. Levels of 27 biomarkers were measured in duplicate using enzyme-linked immunosorbent assay. Spearman correlation coefficients were calculated for the association between biomarker levels or their change on treatment and either bone scan response (BSR) or soft tissue response according to RECIST.

**Results:**

A BSR and RECIST response were seen in 66/81 pts (81 %) and 6/33 pts (18 %) respectively. No significant associations were found between any biomarker at any time point and either type of response. Plasma concentrations of VEGFA, FLT3L, c-MET, AXL, Gas6A, bone-specific alkaline phosphatase, interleukin-8 and the hypoxia markers CA9 and clusterin significantly increased during treatment with cabozantinib irrespective of response. The plasma concentrations of VEGFR2, Trap5b, Angiopoietin-2, TIMP-2 and TIE-2 significantly decreased during treatment with caboznatinib.

**Conclusions:**

Our data did not reveal plasma biomarkers associated with response to cabozantinib. The observed alterations in several biomarkers during treatment with cabozantinib may provide insights on the effects of cabozantinib on tumor cells and on tumor micro-environment and may help point to potential co-targeting approaches.

## Background

Many molecular and cellular adaptations take place within cancer cells and their immediate micro-environment throughout cancer progression to facilitate further proliferation and metastasis. These include adaptation to hypoxia in the tumour microenvironment, epithelial-to-mesenchymal transition (EMT), secretion of pro-inflammatory cytokines and the induction of signaling pathways related to cellular division and invasion [1]. Hypoxia has been recognized as an important poor prognostic factor in prostate cancer [2, 3], associated with increased metastasis formation and chemo-resistance. One of the main mediators of the hypoxic response is the transcription factor HIF-1α [4] that initiates a hypoxia-induced transcriptional program and the subsequent activation of the VEGF-VEGFR pathway (reviewed in [5]). It also leads to expression of carbonic anhydrase-IX (CA9), a membrane-bound protein that maintains intra-cellular pH by catalyzing the extra-cellular conversion of CO_2_ to H^+^ and HCO3^−^ [6]. In addition, the receptor tyrosine kinase c-MET, known to exert a major role in tumor formation and progression, has been shown to be induced in hypoxic cancers in general [7], and in advanced or androgen-receptor-independent prostate cancer in particular [8], especially in bone metastases [9]. MET and VEGFR2 were recently shown to dimerize [10], and VEGF blockade was shown to restore and increase MET activity in GBM cells in a hypoxia-independent manner, while inducing a program reminiscent of EMT [10]. These observations suggest that co-targeting of these receptors may be necessary in order to abrogate their effects on the tumour.

Despite the increase in the armamentarium of active drugs in mCRPC, the disease remains incurable and more therapeutic strategies are needed. Cabozantinib was developed as a dual inhibitor of both MET and VEGFR2 and generated significant interest in the oncology community after it was shown to significantly improve bone scans and alleviate pain in patients with bone-metastatic prostate cancer in a randomized phase II trial, leading to the early termination of the randomization phase of the study [11]. Seventy-two percent of patients had regression in soft tissue lesions, whereas 68 % of evaluable patients had improvement on bone scan, including complete resolution in 12 %. The results of an expansion cohort of the phase II trial (NCT00940225) were recently published [12]. Of 144 patients sequentially enrolled in either a 100-mg (n = 93) or 40-mg (n = 51) study cohort, 91 patients (63 %) had a bone scan response. A reduction in measurable soft tissue disease was also observed in 10 out of 54 patients (19 %).

Here our primary aim was to study the association between plasma concentrations of known markers of hypoxia, cell signaling, inflammation, bone metabolism, chemo-attraction and EMT and response to cabozantinib in a cohort of pts who received the drug at 100 mg daily as part of the non-randomized expansion cohort. Our secondary aim was to study the changes that occur in the levels of these markers on treatment, irrespective of response.

## Methods

### Patients, study design and study assessments

A full description of the patient population, study design, drug administration and study assessments can be found in the manuscript reporting the results of this expansion cohort [12]. Briefly, eligible patients had CRPC and bone metastases on bone scan, all underwent previous treatment with docetaxel and had disease progression during or within 6 months of their most recent standard treatment with a taxane or abiraterone-containing regimen. The clinical study was conducted in compliance with the Declaration of Helsinki and approved by the institutional review boards of participating institutions. Consent for biomarker analysis was obtained from all patients reported herein.

The current post hoc biomarker analysis was performed on blood samples obtained from 81 patients out of the 93 patients of the 100-mg cohort [12], of which 33 had measurable disease (according to RECIST version 1.1) at baseline and at least one post-baseline assessment. We chose to focus on the 100-mg cohort as it was larger, and the responses observed in this cohort were more robust.

Whole-body bone scans and CT scans were acquired at baseline and every 6 weeks until drug cessation. A computer-aided detection system (IBIS, MedQIA, Los Angeles, CA) was used to objectively identify and quantify bone metastases as explained in [13]. After image normalization, the software automatically identified and marked all candidate lesions and calculated the bone-scan lesion area (BSLA). Bone scan response (BSR) was defined as ≥30 % reduction in BSLA between a time point and baseline scan (the full calculation method is described in [13]). For patients with measurable disease, response was assessed using the Response Evaluation Criteria In Solid Tumors (RECIST 1.1), and percent change at each time-point to baseline was calculated. The time of best response was defined individually for either type of response as the time point in which the maximal negative change in percentage was observed or the minimal positive change in percentage was observed.

### Blood samples and biomarker analysis

Blood samples for biomarker analysis were drawn from pts on the trial at baseline and every 6 weeks until either drug cessation or 24 weeks (whichever occurred first). Plasma samples were shipped on dry ice to AssayGate (Ijamsville, MD), and 300 ul of each sample was used for multiplex enzyme-linked immunoabsorbent assay (ELISA) using standard protocols. The experiment was performed in duplicates and the reliability of the duplicate was checked using Pearson correlation. The biomarkers assessed in our current analysis were chosen based on our working hypotheses and/or reported evidence/rationale and included the hypoxia-related markers: Carbonic anhydrase 9 (CA9), GLUT1, Clusterin, Caveolin, Osteopontin; the receptor-ligand pairs: vascular endothelial growth factor (VEGF)-A, VEGF receptor 2 (VEGFR2), hepatocyte growth factor (HGF), c-MET, Fms-related tyrosine kinase 3 (FLT3), FLT3 ligand (FLT3L), insulin-like-growth-factor (IGF) 1 receptor (IGF1R), IGFI, IGFII, AXL, Gas6, stem cell factor (SCF); the inflammation-related markers: c-reactive protein (CRP), interleukin-6 (IL6); the bone-related markers: bone-specific alkaline phosphate (BSAP), Semaphorin-3C (SEMA3C), tartrate-resistant-acid-phopsphatase 5b (Trap5b); and the micro-environment/angiogenesis related markers: tissue inhibitor of matrix metalloprotease 2 (TIMP-2), interlekin-8 (IL-8), thrombospondin-1, angiopoietin-2 (ANG2) and TIE2 (Table 1). Biomarker analysis was performed at baseline, at 6 weeks and at time of best response for each of the two response parameters. If the best response occurred after 24 weeks, the blood sample as 24 weeks was taken instead.

**Table 1:** Plasma markers assessed in the post hoc analysis

Hypoxia-related markers		Signaling pathways		Inflammation		Bone-related markers		Micro-environment/angiogenesis	
Marker	Ref.	Marker	Ref.	Marker	Ref.	Marker	Ref.	Marker	Ref.
CA9	[34]	VEGFA-VEGFR2	[14]	CRP	[35]	BSAP	[12]	TIMP-2	[36]
GLUT1	[37]	HGF- c-MET	[7, 10, 16]	IL-6	[38]	SEMA3C	[39]	IL-8	[40, 41]
Clusterin	[42]	FLT3-FLT3L	[18]			Trap5B	[43]	Thrombo-spondin-1	[44]
Caveolin	[45]	IGF1R-IGFI/IGFII	[20, 46, 47]					ANG2-TIE2	[48, 18]
Osteopontin	[49]	AXL-GAS6	[30, 10, 16]						
		SCF	[18]						

### Statistical analysis

Our primary aim was to determine if associations exist between the best BSR or soft tissue response to any of the 5 following variables: biomarker level at baseline; biomarker level 6 weeks; biomarker level at time of best response; change in biomarker level from baseline to 6 weeks or change in biomarker level from baseline to time of best response. For each biomarker at each time point, two repeats were averaged. The associations between the markers or their change from baseline and the response were evaluated based on the Spearman correlation coefficients.

The changes of the markers over time were explored by applying the mixed effect models to account for the possible correlations between the measurements of the same patient. For these models the outcome was the markers, the covariate was the time and the patient was the random effect. The residuals were inspected from any departure from normality. When the residuals appeared skewed, a transformation was applied to the outcome variable (the marker), which was either log or square root transformation, depending on which made the distribution of the residuals closer to the normal distribution. The type of transformation applied is supplied in the Table 3.

## Results

### Patients and responses

**Fig. 1:**
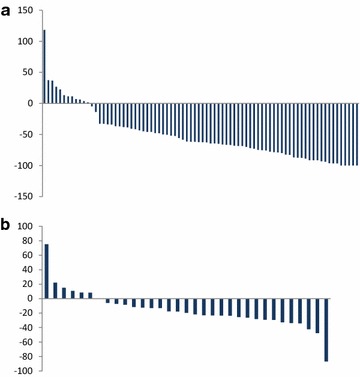
Waterfall plots of **a** BSR and **b** RECIST response of patients treated with cabozantinib at 100 mg a day and were included in this post hoc analysis

The median age of pts included in our study was 67, all had an ECOG PS of 0 or 1, all had metastatic disease to the bone, and all were previously treated with docetaxel, whereas a third were also treated with either enzalutamide, abiraterone or both, and a quarter received prior cabazitaxel. Almost half of the patients had a pain score of 4 or higher (using to the standard 1-10 numeric rating scale). The waterfall plot of the bone scan response of the 81 patients included in this analysis and the soft tissue response of the 33 patients with measurable disease are depicted in Fig. 1, showing that 66 of 81 pts (81 %) had a decrease in BSLA of more than 30 %, and 6 of 33 pts (18 %) had a partial soft tissue response according to RECIST.

### Reliability of measurements

All markers except TIMP2 were reliably measured, with the Pearson correlation coefficient between the two repeats ranging between 0.80 and 0.99. The correlation was lower for TIMP2 at 0.68 (results not shown).

### Correlation of markers with activity

No significant correlation was observed between BSR and marker levels at baseline, 6 weeks or time of best response or the change of the markers from baseline to either 6 weeks or time of best response. The Spearman correlation coefficients ranged between −0.37 and 0.25 (results not shown). When using the Bonferroni p value correction for multiple comparison, no significant correlations were observed between the soft tissue response and marker levels at any time point or the change in marker levels. The Spearman correlation coefficients ranged between: −0.4 and 0.5. The Spearman correlation coefficients with an absolute value of 0.4 or higher (with their corresponding non-corrected p values) are given in Table 2.

**Table 2:** Spearman correlation co-efficients associated with soft tissue response for each of the variables

Variable	Biomarker	Spearman correlation co-efficient	P value (not corrected)
Level of biomarker at baseline	Trap5b	0.45	0.007
Level of biomarker at 6 weeks	Trap5b	0.5	0.002
Level of biomarker at time of best response or earlier	IGF-II	−0.4	0.02
	BoneAP	0.46	0.006
	Trapb5	0.47	0.006
Change in biomarker from baseline to 6 weeks	None		
Change in biomarker from baseline to best response or earlier	TIMP2	0.41	0.02

### Correlation with treatment course

We then assessed trends in marker levels on treatment irrespective of response. Fourteen out of 27 markers showed a significant change in their expression levels throughout treatment, using an alpha level for significance of 0.0018 according to the Bonferroni correction for 27 comparisons (Table 3). The plasma concentration of soluble VEGFR2 was significantly decreased during treatment with cabozantinib, and the plasma levels of VEGF-A were significantly increased, in keeping with the well-characterized biomarker ‘signature’ of VEGFR inhibition [14].

**Table 3:** Change in biomarkers on treatment

	Transformation	Trend	Estimates based on the model					*p* value (not corrected)	Bonferroni adjusted p value
			Baseline	Week 6	Week 12	Week 18	Week 24		
Hypoxia-related makers									
CA9	Square root	Increase	8.39	13.77	15.43	17.22	13.96	0	<0.0001
Clusterin	Log		5.08	5.29	5.18	5.24	5.49	0.00042	0.0088
GLUT1	Log	No significant change	3.9	3.86	3.87	3.87	3.77	0.013	0.35
Caveolin	Square root		2.65	2.51	2.55	2.53	2.27	0.55	>0.99
OPN	Log		4.43	4.23	4.18	4.25	4.49	0.27	>0.99
Signaling pathways									
VEGFA	Log	Increase	3.82	4.94	4.96	4.98	4.7	0	<0.0001
FLT3L	Log		5.31	6.62	6.78	6.51	6.41	0	<0.0001
AXL	None		5452.58	7689.79	7603.41	7055.92	7348.19	0	<0.0001
Gas6	Square root		38.54	55.03	50.85	45.67	52.88	0	<0.0001
c-MET	None		106.04	147.79	142.02	124.13	155.28	7.8E-09	<0.0001
VEGFR	Log	Decrease	7.32	6.66	6.37	6.37	7.02	1.7E-12	<0.0001
FLT3	Log	No significant change	3.98	3.71	3.75	3.91	3.84	0.003	0.081
SCF	Log		4.45	4.36	4.3	4.19	4.72	0.071	>0.99
IGF1R	Log		5.43	5.58	5.6	5.64	5.57	0.035	0.95
IGFI	None		48,276.42	49,765.76	38,327.24	46,418.15	47,612.38	0.034	0.92
IGFII	None		149.85	168.03	151.46	147.71	146.22	0.34	>0.99
HGF	Square root		22.19	18.72	19.45	18.37	22.34	0.06	>0.99
Inflammation									
CRP	Square root	No significant change	91.13	89.21	89.95	94.68	75.03	0.83	>0.99
IL6	Log		2.93	2.9	2.95	2.97	2.99	0.98	>0.99
Bone-related markers									
BSAP	None	Increase	153.54	179.84	175.52	141.5	164.09	0.00024	0.0065
Trap5b	Log	Decrease	1.44	1.16	1.2	1.16	1.31	0.00002	0.0005
SEMA3C	Log	No significant change	4.23	4.45	4.35	3.78	3.8	0.22	>0.99
Micro-environment/angiogenesis									
IL8	Log	Increase	2.18	2.49	2.68	2.68	2.78	1.5E-12	<0.0001
ANG2	Log	Decrease	6.95	6.47	6.48	6.6	6.3	2.5E-08	<0.0001
TIMP2	None		72.28	65.65	66.34	65.08	62.28	1.2E-06	<0.0001
TIE2	Log		8.62	8.25	8.1	7.89	8.43	0.00002	0.0005
Thrombo spondin	Square root	No significant change	64.49	61.72	64.22	49.68	71.9	0.055	>0.99

**Fig. 2:**
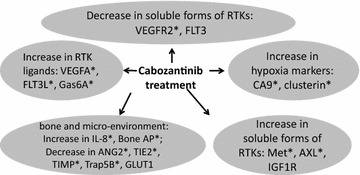
Schematic representation of the significant alterations in plasma biomarkers during treatment with cabozantinib. *Asterisk* signifies a corrected p value (based on the Bonferroni correction for multiple comparisons) <0.05

The plasma concentrations of the soluble forms of the RTKs c-MET and AXL significantly increased upon treatment with cabozantinib. The plasma concentrations of Gas6, FLT3L, Bone-specific alkaline phosphatase and IL-8 also significantly increased upon treatment with cabozantinib irrespective of response. In addition, the plasma concentrations of CA9, a known hypoxia-related marker, and clusterin, a hypoxia-related anti-apoptotic protein, were both significantly increased upon treatment with cabozantinib irrespective of response. In contrast, the plasma concentration of Trap5b, ANG-2, TIMP-2 and TIE2 all significantly decreased following treatment with cabozantinib. A schematic depiction of the alterations in plasma biomarkers during cabozantinib treatment is shown in Fig. 2.

## Discussion

Tyrosine kinase inhibitors have been routinely used in the clinic for treatment of solid and hematological cancers for almost a decade. Tyrosine kinase inhibitors of VEGFR have been used in a variety of solid cancers including kidney, thyroid, liver and recently gastro-intestinal [15]; yet despite much research effort along many years and across many research groups, no predictive biomarkers of response to VEGFR-inhibition have been described to date. Our primary underlying hypothesis in this study was that hypoxia-related markers would be associated with response to cabozantinib; but similar to others, we did not find any significant associations between plasma biomarkers at any time point or their change throughout treatment and either bone scan response or soft tissue response to cabozantinib. A major limitation of our study is the small cohort of patients with measurable disease (n = 33), of which only six patients had a partial response. This cohort size would have allowed only very strong associations between markers and response to reach statistical significance. Our data cannot, at this point, rule out associations of lesser strength that would have become statistically significant with a bigger cohort. Moreover, as the most common type of soft tissue lesion is lymph node metastasis, it is unlikely that a response in lymph nodes would significantly contribute to a change in a serum biomarker. Further work is thus needed in order to elucidate which molecular, clinical or pathological variables determine responsiveness to cabozantinib in prostate cancer.

Our current work does, however, point to significant alterations that occur within the plasma following treatment with cabozantinib irrespective of response. Cabozantinib was rationally designed to inhibit the RTKs VEGFR2 and c-MET. The biological rationale to combine VEGFR2 inhibition with c-MET inhibition is supported by reports describing increased expression or activity of the c-MET tyrosine kinase following inhibition of VEGFR2 [16]. Cabozantinib was shown to result in more extensive anti-tumor activity in animal models than a multi-kinase inhibitor targeting VEGFR2 without c-MET inhibition [17], and to suppress metastasis, angiogenesis and tumor growth across a variety of tumor xenograft models [18]. Our observation of decreased soluble VEGFR2 on treatment with cabozantinib, concomitant with an increase in VEGF-A, is in keeping with the well-characterized biological signature of VEGFR inhibition [14]. In contrast, soluble c-MET and AXL levels were increased on treatment with cabozantinib in our cohort.

A similar pattern of c-MET increase was also reported in patients with progressive/recurrent glioblastoma treated with cabozantinib in a phase 2 trial [19] and in a single-institution subset of patients from this mCRPC patient cohort [20]. The biological significance of the increase in soluble c-MET seen on treatment is currently unclear. In preclinical models, both complete and partial inhibition of c-MET phosphorylation in vivo by cabozantinib has been described [16, 18, 21–24]. A correlative biomarker analysis of patients treated with cabozantinib across several clinical trials showed decreases in phosphorylation of c-Met, AKT and ERK in surrogate hair tissue on drug [25]. In addition, in the single-institution subset of patients from this mCRPC patient cohort described above, phosphorylation of c-MET in metastatic bone lesions was decreased at 6 weeks in 5 of 9 (56 %) patients who had detectable phosphorylation at baseline [20]. The median reduction in phospho-c-MET in that study was 30 %, indicating that the receptor may have been re-phosphorylated and potentially re-activated at 6 weeks. Additional investigations using a subcutaneous CRPC xenograft model in mice revealed that inhibition of c-MET phosphorylation occurred early following the administration of cabozantinib, but was followed by an increase in the phospho-c-MET signal at a later time point, perhaps as a result of non-ligand induced re-phosphorylation of the receptor [26]. Further research is clearly needed to fully characterize the nature, extent and duration of the effect of cabozantinib on c-MET phosphorylation and/or signaling in prostate cancer in vivo.

The hypoxia-related markers CA-9 and clusterin significantly increased following treatment with cabozantinib, suggesting modulation of tumour hypoxia or the response thereto in the presence of cabozantinib. This is in line with the recent observation that cabozantinib increases hypoxia in medullary thyroid cancer cells by modulating HIF1 [27]. Our analysis did not reveal a statistically significant association between the increase in hypoxia-related markers and response, but it is currently unknown whether there is a significant association between cabozantinib-induced-hypoxia and time to tumor progression. The crosstalk between RTK inhibition, the induction of micro-environmental hypoxia and tumour evolution should be further studied.

In addition to VEGFR2 and c-MET inhibition, cabozantinib also inhibits other RTKs in vitro, including RET, KIT, AXL and FLT3 [18, 28]. FLT3 levels non-significantly decreased on treatment and FLT3L levels significantly increased on treatment, similar to the pattern observed for the VEGFR2-VEGF-A pair. In contrast, both the levels of soluble AXL receptor and the AXL ligand Gas6A significantly increased on treatment. AXL was shown to be highly expressed in metastatic prostate cancer and its interaction with Gas6 was suggested to play a role in establishing tumor dormancy in the bone marrow microenvironment [29]. AXL promotes migration and invasion of prostate cancer cells in vitro and regulates expression of genes involved in EMT. Gas6 negatively regulates AXL expression levels in general, but not in hypoxic environments such as in a tumor or in bone [30]. It is tempting to speculate that the observed increase, rather than the expected decrease, in soluble plasma AXL levels is a manifestation of its increased expression in cancer cells that is in turn a result of cabozantinib-induced-hypoxia. This may imply that the potential beneficial effects of AXL inhibition by cabozantinib are mitigated by the concomitant increase in hypoxia. In medullary thyroid cancer, inhibition of cabonzatinib-induced hypoxia by the HIF-1 inhibitor 2-methoxyestradiol enhanced the drug’s efficacy in vitro and in vivo [27]. Clearly more work is needed in order to study the effects of caboznatinib on hypoxia and on the Gas6-AXL pathway, and the relationship of both to prostate cancer progression.

Additional alterations were shown to occur following treatment with cabozantinib. The levels of TIMP2 and TIE2 were decreased on cabozantinib; this is in line with the observed decrease in their levels in patients with renal cell carcinoma treated with the multi-VEGFR-PDGFR inhibitor regorafenib [31], demonstrating a consistent change in micro-environment-related and angiogenesis-related biomarkers on treatment with VEGFR TKI.

Recently, the results of the phase III trials of cabozantinib in mCRPC were presented, failing to demonstrate a statistically significant overall survival benefit vs. placebo, or a palliative benefit for cabozantinib vs. mitoxantrone/prednisone in heavily pre-treated mCRPC patients ([32] and [33], respectively). The promising response rates observed in the phase II trials therefore did not translate into an OS benefit for the entire cabozantinib-treated population. Indeed, concerns have been raised that the dramatic bone scan response seen following treatment with caboznatinib are the result of non-specific effects on bone turnover rather than a true anti-neoplastic effect within that niche [50].

The results presented here show that cabozantinib induces significant changes in several plasma biomarkers known to be linked to hypoxia, tumor micro-environment and RTK signaling. It will be interesting to see if these significant alterations are associated with other endpoints of clinical importance such as time to progression and overall survival. Further basic, translational and clinical research on these alterations may enhance our understanding of the mechanism of action and of cabozantinib as well as mechanisms of drug resistance and may point to potential co-targeting approaches. Our current work may thus inform ongoing approved and emerging indications for cabozantinib.

## Conclusions

Whereas our work did not find plasma biomarkers associated with response to cabonzatinib in mCRPC, it does point to plasma biomarkers that are significantly altered upon treatment with the drug. These include the receptor ligand pairs MET-HGF, VEGFR2-VEGF-A, FLT3-FLT3L and AXL-GAS6, the hypoxia-related markers CA-9 and clusterin and the micro-environmental factors TIMP2 and TIE2, suggesting that these molecular players and pathways play a role in the tumor, the micro-environment and the systemic response to cabozantinib. Further research on the relationship between the alteration in these signaling pathways, response/resistance to cabozantinib and tumor progression is clearly warranted.

## Abbreviations

ANG2: angiopoietin-2
BSAP: bone-specific alkaline phosphate
BSR: bone scan response
CA9: carbonic anhydrase 9
CRP: c-reactive protein
ELISA: enzyme-linked immunoabsorbet assay
EMT: epithelial-mesenchymal transition
FLT3: Fms-related tyrosine kinase 3
FLT3L: FLT3 ligand
HGF: hepatocyte growth factor
IGF1R: insulin-like-growth-factor 1 receptor (IGF1R)
IGFI: insulin growth factor I
IGFII: insulin growth factor II
IL-6: interleukin-6
IL-8: interlekin-8
mCRPC: metastatic castration resistant prostate cancer
RECIST: response evaluation criteria in solid tumors
SEMA3C: semaphorin-3C
SCF: stem cell factor
Trap5b: tartrate-resistant-acid-phopsphatase 5b
TIMP-2: tissue inhibitor of matrix metalloprotease 2
VEGFA: vascular endothelial growth factor A
VEGFR: vascular endothelial growth factor recptor 2
